# First Investigation of Haemosporidian Species and Record of Novel Genetic Lineages in Eurasian Griffon Vultures (*Gyps fulvus*) in Greece

**DOI:** 10.3390/vetsci12100973

**Published:** 2025-10-10

**Authors:** Grigorios Markakis, Vaidas Palinauskas, Justė Aželytė, Isaia Symeonidou, Aurelija Krumplevskaja, Anastasia Komnenou, Elias Papadopoulos

**Affiliations:** 1 School of Veterinary Medicine, Faculty of Health Sciences, Aristotle University, 54124 Thessaloniki, Greece; grigmark@vet.auth.gr (G.M.); natakomn@vet.auth.gr (A.K.); eliaspap@vet.auth.gr (E.P.); 2 ANIMA—Association for Wildlife Care and Protection, 10442 Athens, Greece; 3 State Scientific Research Institute Nature Research Centre, 08412 Vilnius, Lithuania; vaidas.palinauskas@gamtc.lt (V.P.); juste.azelyte@gamtc.lt (J.A.); aurelija.krumplevskaja@gmc.stud.vu.lt (A.K.)

**Keywords:** haemosporidians, Eurasian griffon vultures, *Leucocytozoon*, *Haemoproteus*, *Plasmodium*, new genetic lineages

## Abstract

**Simple Summary:**

Blood parasites can harm the health of wild birds, yet little is known about the blood parasites of Eurasian griffon vultures, a scavenger bird species that is endangered in many parts of the world, including Greece. This study examined 59 griffon vultures admitted to a wildlife rescue center in Greece. Blood samples were analyzed using microscopy and genetic techniques to detect three specific groups of blood parasites called Haemosporidians. The overall infection rate was relatively low, suggesting that griffon vultures may possess a strong immune response against such infections. However, the study identified several previously unrecorded genetic types of Haemosporidians in vultures. These findings expand our understanding of parasite diversity in this species and highlight the importance of further research to clarify the impact of these parasites on vulture population dynamics, which is useful for developing effective conservation strategies.

**Abstract:**

Eurasian griffon vultures (*Gyps fulvus*) are endangered scavenger birds. Haemosporidian parasites infect the blood cells and organs of many avian species globally, using blood-sucking insect vectors, and they negatively affect health of birds and subsequently wildlife biodiversity. Fifty-nine vultures were admitted to the Greek wildlife rehabilitation center “ANIMA” and included in this study. Blood samples were collected, and the microscopy of stained blood smears was performed. Moreover, DNA was extracted, samples were screened for *Leucocytozoon*, *Haemoproteus*, and *Plasmodium* spp. following nested PCR protocols, and positive samples were sequenced. The detected haemosporidians are reported for the first time in Eurasian griffon vultures globally. The overall prevalence was 11.9% (*Leucocytozoon* spp. 5.1%, *Haemoproteus* spp. 5.1%, *Plasmodium* spp. 3.4%); this further corroborates the statement that the vultures’ immunity protects them from blood parasites. Notably, new genetic lineages of *Leucocytozoon (GYPFUL02)*, *Haemoproteus* (*GYPFUL01*), and *Plasmodium* (*GYPFUL03*) species were recorded for the first time. Furthermore, it was the first molecular isolation of *Haemoproteus* in Old World vultures and *Haemoproteus brachiatus* was isolated for the first time from a vulture species. Moreover, this demonstrates the first record of *Parahaemoproteus* genetic lineages in vultures. Results highlight the genetic diversity of haemosporidians in griffon vultures and the need for additional studies.

## 1. Introduction

The scavenger birds, feeding on environmentally decaying organic material, are thought to be disease moderators, hence they play a vital role in the ecosystems [1,2]. Vultures belong to this group as well as to the category of the most endangered avian species worldwide; from the 22 vulture species, most of them have declining populations and are in danger of extinction [1]. Greece hosts 4 species of vultures, these are: the Eurasian griffon vulture (*Gyps fulvus*), the Cinereous vulture (*Aegypius monachus*), the bearded vulture (*Gypaetus barbatus*), and the Egyptian vulture (*Neophron percnopterus*) [3]. Eurasian griffon vultures are obligate avian scavengers of the Old World, constituting the most numerous and gregarious European vulture species (Vulture Conservation Foundation) [2]. They reside in Greece, with only occasional migrations by juveniles [4] and, noteworthily, in this country they are considered critically endangered (International Union for Conservation of Nature).

Griffon vultures are hosts for many parasites. Haemosporidians are a wide group of vector-borne protozoan parasites that infect the blood cells and other tissues of most avian species worldwide and are transmitted via blood-sucking dipteran insects [5]. Species of the genera *Leucocytozoon*, *Haemoproteus*, and *Plasmodium* are the most extensively studied and they are considered the most important regarding wild birds [5,6]. They are reported to have a detrimental effect on birds’ health, especially on debilitated hosts, and severe clinical signs such as anemia, diarrhea, dyspnea and even lameness and neurological symptoms have been recorded in wild avifauna [7,8,9]. Regarding vultures, a decrease in body condition score in cinereous vultures infected by haemosporidians has recently been demonstrated [10].

Parasitism of vultures by haemosporidians has barely been studied. Until recently, blood parasites of vultures have only been studied by microscopy, which is less sensitive for defining diversity of parasites and prevalence during light infections than the Polymerase Chain Reaction (PCR)-based methods [10]. It is estimated that these birds have a low occurrence of blood parasites due to their enhanced immune system [11,12]. To better understand the complexities of these parasites in vulture populations, further studies should investigate the relationship between vulture immunity, nesting habits, and Haemosporidian infections. However, infections with haemosporidians in conjunction with the strong human-related pressure that these birds are subjected to (e.g., medicine used in livestock, intoxications, etc.) could put at risk the future of these species [10,13,14,15,16]. The aim of this study was to assess, for the first time, the prevalence rate and the genetic diversity of infection with haemosporidians in Eurasian griffon vultures in Greece.

## 2. Materials and Methods

### 2.1. Sample Collection–Microscopical Examination

Fifty-nine (59) Eurasian griffon vultures that were admitted to “ANIMA, Association for Wildlife Care and Protection” in Athens (Greece) were involved in this study. All of these birds originated from Heraklion (*n* = 26), Chania (*n* = 18) and Rethymno (*n* = 15), three geographical departments of the island of Crete, and they were all subadult individuals presenting with low body condition score and dehydration. The sample collection was performed during the summer and autumn of 2023 and 2024. In detail, blood samples (100 μL) were collected from all vultures instantly after their admission by puncturing the brachial vein. Blood smears were prepared, air dried, fixed with methanol and then stained with Giemsa for morphological examination [17]. The smears were examined by a skilled parasitologist who tested 100 microscopic fields at low magnification (×400), and then 100 fields at high magnification (×1000) using a Motic BA210 microscope. Overall, approximately 5 × 10^5^ red blood cells were screened in each blood film [17].

### 2.2. Molecular Analysis

The blood was preserved in a SET buffer (0.015 M NaCl, 0.05 M Tris, 0.001 M ethylenediaminetetraacetic acid, pH 8, 1:10) and then stored at −20 °C. DNA was extracted following the Quick-DNATM Miniprep Kit by Zymo Research.

Blood samples were analyzed for haemosporidian parasites using two nested PCR protocols. First, all samples were screened following a modified nested PCR protocol [18] by Perez-Rodriguez et al. [19], which amplifies a fragment of the mitochondrial cytochrome b gene of *Leucocytozoon* spp., *Haemoproteus* spp. and *Plasmodium* spp. This protocol consists of a first PCR step using the primers Plas1F (5′-GAGAATTATGGAGTGGATGGTG-3′) [20] and HaemNR3 (5′-ATAGAAAGATAAGAAATACCATTC-3′) [21], and afterwards a nested PCR step with the internal primers 3760F (5′-GAGTGGATGGTGTTTTAGAT-3′) [22] and the HaemJR4 (5′-GAAATACCATTCTGGAACAATATG-3′), which amplify a cyt b fragment suitable for parasite detection across all three genera.

To improve lineage identification and resolve potential mixed infections, especially those involving *Leucocytozoon* spp.—all samples that tested positive in the first protocol were subsequently amplified using a second nested PCR approach following the protocol described by Hellgren et al. [21], which specifically targets *Haemoproteus* and *Plasmodium* lineages. The outer primers were HaemNFI (5′-CATATATTAAGAGAAITATGGAG-3′) and HaemNR3, and the nested PCR used HaemF (5′-ATGGTGCTTTCGATATATGCATG-3′) and HaemR2 (5′-GCATTATCTGGATGTGATAATGGT-3′) primers [21,23]. DNA isolated from a previously identified *Plasmodium relictum* parasite was used as the positive control, while nuclease-free water was added as the negative control instead of the sample DNA. The combined use of these two nested PCR protocols enabled broad detection of haemosporidian genera and precise lineage identification, with primers specifically chosen for their suitability in raptorial birds and effectiveness in detecting mixed infections [19,21].

The PCR positive products were sequenced using Big Dye Terminator V3.1 Cycle Sequencing Kit and ABI PRISMTM 3100 capillary sequencing robot (Applied Biosystems, Foster City, CA, USA). The obtained sequences were trimmed and aligned using Geneious software (version 2024.0.7). The genetic lineages of haemosporidian parasites were compared with those available in the NCBI GenBank database, and matches were also searched for in the MalAvi database of avian haemosporidian parasites [24]. Sequence similarity searches were performed using the BLAST+ 2.16.0 algorithm.

### 2.3. Phylogenetic Analysis

Phylogenetic analysis was performed using a total of 32 sequences, including 28 from previously identified genetic lineages retrieved from the MalAvi database and one *Plasmodium falciparum* sequence obtained from GenBank, which was used as an outgroup. Additionally, three newly identified genetic lineages from this study were incorporated into the analysis. Sequence assembly, editing, alignment, and calculation of pairwise genetic distances were conducted in Geneious software (version 2024.0.7). All sequences were trimmed to a standardized length of 478 base pairs. The optimal evolutionary model for phylogenetic reconstruction was selected using jModelTest (version 2.1.10) [25], with the GTR + I + G model identified as the best fit for the tree. A phylogenetic tree was constructed using Bayesian methods implemented in MrBayes (version 3.2.7a) [26]. The analysis was run for 10 million generations with sampling every 100th generation, and the first 25% of sampled trees were discarded as burn-in.

### 2.4. Statistical Analysis

The overall prevalence and the prevalence per studied haemosporidian parasite were calculated by dividing the number of infected birds by the total number of birds sampled; the 95% confidence intervals were estimated using the Wilson score method (Epitools, ©Ausvet, Fremantle, Australia, 2025).

## 3. Results

**Figure 1:**
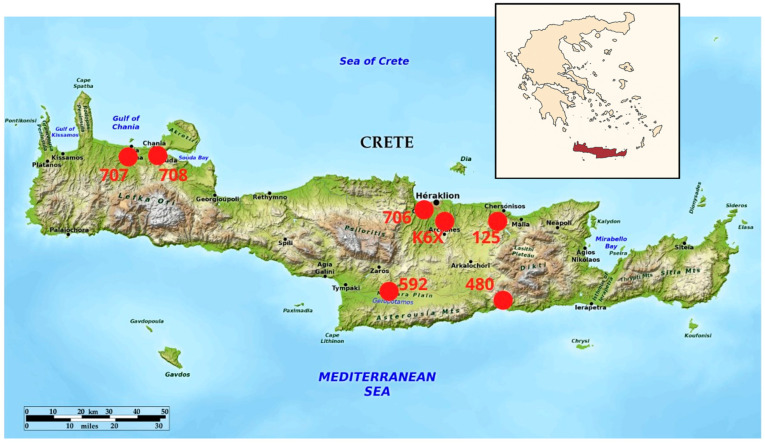
The exact spots in the island of Crete (Greece) where the infected Eurasian griffon vultures were detected with the provided ID numbers of the birds.

Seven out of the fifty-nine Eurasian griffon vultures tested positive for haemosporidian parasites using molecular methods. The overall prevalence of haemosporidian parasites in the studied population was 11.9% (7/59; 95% CI, 5.9–22.5%). The prevalence of infections by *Leucocytozoon* spp., *Haemoproteus* spp. and *Plasmodium* spp. were 5.1% (3/59; 95% CI, 1.7–13.9%), 5.1% (3/59; 95% CI, 1.7–13.9%), and 3.4% (2/59; 95% CI, 0.9–11.5%), respectively. The region of origin and the infection status of the PCR-positive griffon vultures are demonstrated in Table 1. The exact spots where the infected birds were detected are depicted in Figure 1. No haemosporidian parasites were detected in blood smears by microscopy. However, since parasitemia intensity and smear quality affect the detection sensitivity, the presence of blood stages of parasites cannot be conclusively excluded.

**Figure 2:**
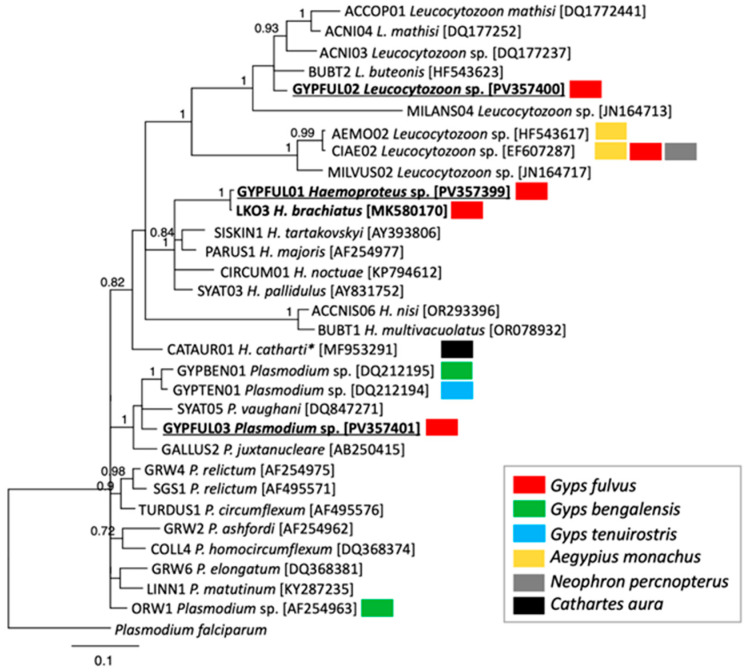
Bayesian inference phylogeny of 32 haemosporidian cytochrome b gene sequences, including 28 from previously identified genetic lineages retrieved from the MalAvi database, three newly recorded lineages, and *Plasmodium falciparum* sequence obtained from GenBank, which was used as an outgroup. The provided MalAvi lineage code is followed by the species name (where available) and the GenBank accession number in parenthesis. All genetic lineages obtained in this study are bold; additionally, new genetic lineages are underlined. The color boxes represent different vulture species and indicate next to the genetic lineages which lineages were recorded in these species in previous molecular studies. Posterior probabilities > 70 are indicated on the tree. * The lineage referred to *H. cathartic*—a parasite which requires additional analysis for its taxonomic clarification [27].

Phylogenetic analysis based on cyt b sequences (Figure 2) revealed four distinct haemosporidian genetic lineages—three novel lineages and the known *H. brachiatus* lineage LK03 (Table 1). The three previously undescribed lineages here are designated as GYPFUL01 (GenBank accession no. PV357399), GYPFUL02 (GenBank accession no. PV357400), and GYPFUL03 (GenBank accession no. PV357401). The novel *Haemoproteus* lineage GYPFUL01, clustered with *H. brachiatus* (lineage LK03) with high support (posterior probability = 1.0) and showed 0.4% sequence divergence from LK03. A newly recorded lineage was obtained after using Hellgren et al. primers [21] (Table 1). In addition, another *Leucocytozoon* sp. was detected in the same individual with Perez-Rodriguez et al. (2013) primers [19]; however, the sequence contained a deletion, making it unsuitable for submission to GenBank or MalAvi databases. The PCR and sequencing were repeated twice, but the result was the same. Another vulture was infected with the known *H. brachiatus* lineage LK03, confirming the presence of parasites from this distinctive clade in griffon vultures. The novel *Leucocytozoon* lineage GYPFUL02 grouped within the *L. toddi* clade, with 6% divergence from *L. buteonis* (lineage BUBT2), and clustered with lineages previously reported from raptors. The novel *Plasmodium* lineage GYPFUL03 found in one griffon vulture from Heraklion, phylogenetically grouped with *Novyella* parasite *P. vaughani* (lineage SYAT05) and two *Plasmodium* spp. lineages, GYPBEN01 and GYPTEN01, which were found in white-rumped vultures (*Gyps bengalensis*) and slender-billed vultures (*Gyps tenuirostris*) in Southeast Asia, respectively (38; Figure 2). Nevertheless, although these lineages cluster within the same clade, the genetic divergence between GYPFUL03 and SYAT05, GYPBEN01, and GYPTEN011 remains appreciable (>4%).

In three vultures from Heraklion, mixed infections were detected using the Pérez-Rodriguez et al. primers [19], but lineage identification was not possible due to overlapping sequence peaks in the electropherograms. BLAST analysis revealed that infection in one vulture corresponded to parasites of the genus *Haemoproteus*, another matched *Leucocytozoon*, and the third matched *Plasmodium* (Table 1). However, in all three cases, the co-infecting parasite could not be resolved with confidence. In another vulture, Pérez-Rodriguez et al. primers [19] amplified *Leucocytozoon* DNA, whereas the Hellgren et al. primers [21] detected *Haemoproteus* (lineage GYPFUL1), confirming a mixed infection. Finally, in one sample, a positive PCR signal was obtained using Hellgren et al. primers [21]; however, sequencing was unsuccessful despite repeated attempts.

## 4. Discussion

The haemosporidian parasites detected in our study are reported for the first time in Eurasian griffon vultures worldwide. Using molecular methods, the total prevalence of infection by the three major genera (*Leucocytozoon*, *Haemoproteus* and *Plasmodium*) was 11.9% (7/59), including three new genetic lineages (GYPFUL01, GYPFUL02, and GYPFUL03). This information contributes significantly to the limited body of knowledge on blood parasites in Old World vultures. The observed prevalence might seem to be low when compared to studies in other bird species, like raptors, in which the molecularly detected prevalence of infection exceeded 50% and, in some cases, reached over 90% [28,29,30]. Nevertheless, the findings of the present study exceed prior molecular studies on vultures, such as a recent survey by Chakarov and Blanco, in which the prevalence of infection was only 3.1% in griffon vultures in Spain [10].

In the current study, no haemosporidians were detected microscopically. Likewise, earlier large-scale surveys from the Iberian Peninsula using microcopy also failed to detect any haemosporidian infections in griffon vultures [31,32]. These discrepancies might reflect the limited sensitivity of microscopy at very low parasitemia, where molecular methods are more effective. Additionally, the possible insufficient quality of the blood smears might further limit the detection rate even for the highly experienced specialist [17]. Another possible explanation could be that not all parasites of detected genetic lineages complete their life cycle in vultures and develop transmissive stages—visible in smears gametocytes—or do so at extremely low levels, rendering them undetectable via microscopy, although DNA of the parasite can be detected. Such cases of abortive development have been reported in previous studies in other avian species [33]. The above limitations in detecting haemosporidian infections by microscopy may also be the case in the current study.

The overall low prevalence of haemosporidian infections in vultures could be explained by the hypothesis that the potency of their immune response seems to protect them from blood parasites [11,12]. The low occurrence of parasitosis in the griffon vultures encountered in our study could also be attributed to the fact that these birds make their nests on barren cliffs, where the contact with the dipteran vectors is limited. Usually, the vectors of haemosporidians look for their hosts in areas with higher humidity and vegetation. This hypothesis was confirmed by a survey conducted in South Africa, where whilst the cliff-nesting Cape vultures (*Gyps coprotheres*) presented no blood parasites [34], on the contrary, the prevalence of haemosporidian infection of some tree-nesting African vulture species was much higher (>30%) [34]. In addition, Chakarov and Blanco found a significantly higher prevalence of *Leucocytozoon* infection in tree-nesting cinereous vultures (*Aegypius monachus*, 10.3%) compared to cliff-nesting species such as griffon vultures (3%) and Egyptian vultures (*Neophron percnopterus*, 5.3%) [10]. Interestingly though, whereas the current study was focused on a cliff-nesting species, the overall prevalence of haemosporidian infection in our sample exceeded 11%, a value notably higher than that reported in prior studies. This discrepancy may be attributed to various factors such as regional variation in vectors’ abundance, differences in sampling time and year, and improvements in molecular detection. This is in accordance with a recent study on New World vultures, which demonstrated that the infection prevalence can vary markedly depending on the vulture species, the locality, and even the year of sampling [27]. In that study, *Haemoproteus catharti* was found exclusively in turkey vultures (*Cathartes aura*), with a 24% overall infection rate, while black vultures (*Coragyps artratus*), despite their close phylogenetic, ecological, and behavioral similarity, presented 0% prevalence. Furthermore, infection rates in turkey vultures varied significantly depending on the region and year, ranging from 33% in eastern U.S. states to only 2% in western states, and from 42% in 2013 to 21% in 2014. Consequently, it appears that haemosporidian infection dynamics in vultures are influenced by a complex interplay of the host species’ identity, geographic locality, habitat type, and temporal factors, underscoring the importance of accounting for these variabilities when comparing the prevalence across different studies.

In the present survey, one of the most prevalent genera detected in the griffon vultures was *Leucocytozoon* (5.1%). Specifically, three infected individuals were detected, one infected by a new genetic lineage of *Leucocytozoon* sp. (GYPFUL02) and the other two with mixed infections by *Leucocytozoon* spp. (Table 1). Phylogenetically, the novel lineage GYPFUL02 clusters within the *L. toddi* clade, which contains lineages previously detected in a wide range of raptors [35], suggesting that parasites infecting griffon vultures are not vulture-specific, but instead belong to a broader raptor-associated lineage group. The only haemosporidian parasite lineage that had been isolated from Eurasian griffon vultures to date was the lineage CIAE02 of *Leucocytozoon* sp. [10], which was not detected in this study. Considering other vulture species, *Leucocytozoon* species of these birds are generally not highly diverse. In cinereous vultures, the CIAE02 and AEMO02 lineages have been detected, while the CIAE02 lineage has also been found in Egyptian vultures (Figure 2) [10]. *Leucocytozoon toddi* has been reported in 0.8% of the South African Old World vulture species [34] and in 0.5% of the Florida’s black vultures (New World species) as well [36]. In general, species of the genus *Leucocytozoon* are considered to be cosmopolitan, as they are found in countries with both hot and cold climates, being a global threat for many avian species, which usually present high prevalence of infection [9]. This is attributable to the black flies that transmit *Leucocytozoon* spp. parasites [9]. Peer-reviewed evidence confirms their occurrence in other regions of Greece [37], suggesting that the ecological and environmental conditions in Crete are also permissive for vector establishment, though formal entomological records in scientific literature of these vectors from Crete are currently lacking. Consequently, there is a credible potential for the transmission of the *Leucocytozoon* species, including the aforementioned newly isolated genetic lineages. However, despite the fact that the black flies can cover large distances, they usually seek birds close to water bodies, and this could explain the relatively low prevalence of infection in the barren cliff-nesting Eurasian griffon vultures. Adding to this, although the nestlings generally present a higher likelihood of being infected due to their thinner plumage, which makes it easier for the vectors to feed on, the contact between the vultures’ nestlings and the vectors may be reduced because of the early breeding of griffon vultures, which results in a discrepancy between their chicks and the peak of the increased activity of the black flies. On the other hand, though, the nestlings of the vultures remain in the nest for a long enough time to be infected before they fledge and, either way, even the adult griffon vultures present larger areas of bare skin for vectors to bite compared to many other raptorial species [10].

The prevalence of *Haemoproteus* spp. infection in the current study was 5.1%. This marks the first molecular documentation of this genus in griffon vultures, and more broadly in any European Old World vulture species. Until now, *Haemoproteus* in Old World vultures had only been detected and described morphologically in African species (*Haemoproteus janovyi* and *Haemoproteus elani*) with no associated molecular data [27,34]. In USA, 24% of the turkey vultures—a New World vulture species—were found positive for *Haemoproteus catharti* [27]. Interestingly, this parasite seems to be evolutionarily distinct from other *Haemoproteus* species, showing closer phylogenetic affinity to reptilian *Haemocystidium* and *Plasmodium* species from birds and reptiles, suggesting paraphyly within *Haemoproteus* [27,35]. This highlights the need for additional studies into the genetic diversity of haemoproteids, which could help redefine the borderlines between the genera of these parasites and clarify the evolutionary relationships among haemosporidian parasites across vertebrate hosts. Moreover, it is notable that the known lineage LK03 of *Haemoproteus brachiatus* and a novel lineage GYPFUL01—phylogenetically related to LK03—were both found in the samples of this survey (Figure 2). This was the first time that a *Haemoproteus brachiatus* genetic lineage, which was previously recorded in birds belonging to *Falco*, *Ninox*, *Otus* and *Circus* genera, was isolated from a vulture species. Moreover, this finding demonstrates the first record of *Parahaemoproteus* genetic lineages in vultures. Its detection in griffon vultures expands the known host range and suggests that certain *Parahaemoproteus* parasites may circulate more broadly among the raptors than previously recognized. *Haemoproteus brachiatus* was previously molecularly characterized by Valkiunas et al. (2019) [38] from the common kestrel (*Falco tinnunculus*). It is noteworthy that the vectors (Ceratopogonidae, Hippoboscidae) of *Haemoproteus* spp. [39] have been recorded in Crete [40,41], underscoring a potential for transmission of the genetic lineages that were identified in the present study.

In the present study, 3.4% of the Eurasian griffon vultures were positive for *Plasmodium* spp., and the novel lineage GYPFUL03 was identified. This represents the first molecular evidence of *Plasmodium* infecting European griffon vultures. In other vulture species, *Plasmodium fallax* has been detected in 0.6% of South African Old World vultures [34], and *Plasmodium elongatum* was identified in 0.5% of black vultures in Florida [36]. Our detection of *Plasmodium* in European griffon vultures challenges the notion that these parasites are entirely absent from this species. Furthermore, the detection of this novel lineage in Greece, together with previous reports *Plasmodium* lineages (e.g., GYPTEN01 and GYPBEN01) in Southeast Asia (Figure 2) [35,42] indicates that malaria parasites may be more widely distributed among vultures than previously assumed. Notably, earlier studies on European Old vulture species failed to detect *Plasmodium* infections [10,31,32]. Therefore, the findings of the current study reinforce the idea that some *Plasmodium* lineages may be more widespread among *Gyps* species than was previously thought. Moreover, it is noteworthy that the presence of *Culicidae* mosquitoes, which are the established vectors of avian malaria, has been reported in the island of Crete [43]. Consequently, on the one hand, the possibility of transmission of lineages is highlighted and on the other hand, the low prevalence of *Plasmodium* spp. infection of the present study can be attributed to other factors. The latter could support the hypothesis that European Old World vultures eventually develop a kind of resilience to infections by *Plasmodium* spp., or, conversely, infections by species belonging to this genus might be fatal, resulting in the non-sampling of infected birds. However, additional larger-scale studies are needed to better assess the prevalence of *Plasmodium* spp. in Eurasian griffon vultures.

The results of the current study provide valuable information about the diversity of the haemosporidian infections in Eurasian griffon vultures, and more broadly, in diurnal raptors. They add to the growing body of literature emphasizing the role of molecular tools in uncovering cryptic parasite diversity, particularly in host groups like vultures. However, it is equally important to highlight that molecular detection solely cannot confirm the completion of the parasite life cycle in the host. Microscopy remains essential for verifying whether the detected lineages reach the gametocyte stage and are therefore potentially transmissible. Targeted investigations, such as this one and others being even more focused on particular parasites, can help resolve taxonomic uncertainties—such as those surrounding *H. catharti*—and improve the identification of widely distributed but morphologically indistinct lineages like CIAE02. Furthermore, this survey stresses the need for studies including tissue histology, which are essential to clarify the developmental biology and transmission potential of these lineages.

The sex of the vultures that took part in the current study was not determined, as vultures do not present the sexual dimorphism that most raptors do. In the same context, the age could not be assessed as a risk factor in the present study, as all the vultures that were involved were subadults. Nevertheless, no effect of bird’s sex or age on the prevalence of haemosporidian infection has been detected for griffon vultures in a large-scale survey [10].

Given the anthropogenic pressures—including habitat fragmentation, food scarcity, and poisoning—that griffon vultures face, haemosporidian infections could pose an additional conservation concern for these ecologically important birds. Improved knowledge of parasite diversity, transmission ecology, and host susceptibility is therefore crucial, not only for understanding the parasite evolution but also for developing informed conservation strategies.

## 5. Conclusions

The detected haemosporidians are reported for the first time in Eurasian griffon vultures globally. Moreover, the current study represents the first investigation of haemosporidian infections in Eurasian griffon vultures in Greece. An overall infection prevalence of 11.9% and new genetic lineages from each of the three haemosporidian genera—*Leucocytozoon*, *Haemoproteus*, and *Plasmodium*—were reported. Particularly notable is the first molecular evidence of *Haemoproteus* infection in an Old World vulture species, including *Haemoproteus brachiatus* (lineage LK03), and its closely related novel lineage GYPFUL01. Moreover, two additional novel lineages—GYPFUL02 of *Leucocytozoon* sp. and GYPFUL03 of *Plasmodium* sp.—were detected. These results expanded the understanding of the geographical distribution of the avian haemosporidians. Future work should aim to clarify the parasites’ life cycle, vector associations, and the pathogenic potential in vultures, with a view towards the assessment of their possible impact on vulnerable populations.

## Figures and Tables

**Table 1:** The region of origin in Crete, the infection status, the obtained parasite lineages depending on the primers employed and Genbank accession numbers of the PCR-positive Eurasian griffon vultures.

*Gyps fulvus* ID	Region of Origin	Single/Mixed Infection	Primers by [19]	Primers by [21]	Genbank Accession Number
592	Heraklion	Mixed	*Leucocytozoon* sp. *	*Haemoproteus* sp. GYPFUL01	PV357399
K6X	Heraklion	Mixed	Mixed (with *H*. sp.) ***	Neg	-
706	Heraklion	Mixed	Mixed (with *P*. sp.) ***	Neg	-
480	Heraklion	Mixed	Mixed (with *L*. sp.) ***	Pos **	-
708	Chania	Single	*Leucocytozoon* sp. GYPFUL02	Neg	PV357400
707	Chania	Single	*Haemoproteus brachiatus* LK03	Neg	MK580170
125	Heraklion	Single	*Plasmodium* sp. GYPFUL03	*Plasmodium* sp. GYPFUL03	PV357401

* The sequence of *Leucocytozoon* sp. was received with a deletion and could not be deposited in GenBank. The whole process (PCR and sequencing) was repeated, and the result was the same. ** Positive according electrophoresis, but no sequence obtained. *** Mixed infection cases where BLAST results showed a strong match to parasites of the genera *Haemoproteus* (*H*. sp.), *Plasmodium* (*P*. sp.), or *Leucocytozoon* (*L*. sp.) but the co-infecting parasite could not be identified reliably.

## Data Availability

The original contributions presented in the study are included in the article. Further inquiries can be directed to the corresponding author(s).
